# Induction of Blood Brain Barrier Tight Junction Protein Alterations by CD8 T Cells

**DOI:** 10.1371/journal.pone.0003037

**Published:** 2008-08-22

**Authors:** Georgette L. Suidan, Jeremiah R. Mcdole, Yi Chen, Istvan Pirko, Aaron J. Johnson

**Affiliations:** 1 Neuroscience Department, University of Cincinnati College of Medicine, Cincinnati, Ohio, United States of America; 2 Neurology Department, University of Cincinnati College of Medicine, Cincinnati, Ohio, United States of America; Institut de la Vision, France

## Abstract

Disruption of the blood brain barrier (BBB) is a hallmark feature of immune-mediated neurological disorders as diverse as viral hemorrhagic fevers, cerebral malaria and acute hemorrhagic leukoencephalitis. Although current models hypothesize that immune cells promote vascular permeability in human disease, the role CD8 T cells play in BBB breakdown remains poorly defined. Our laboratory has developed a novel murine model of CD8 T cell mediated central nervous system (CNS) vascular permeability using a variation of the Theiler's virus model of multiple sclerosis. In previous studies, we observed that MHC class II^−/−^ (CD4 T cell deficient), IFN-γR^−/−^, TNF-α^−/−^, TNFR1^−/−^, TNFR2^−/−^, and TNFR1/TNFR2 double knockout mice as well as those with inhibition of IL-1 and LTβ activity were susceptible to CNS vascular permeability. Therefore, the objective of this study was to determine the extent immune effector proteins utilized by CD8 T cells, perforin and FasL, contributed to CNS vascular permeability. Using techniques such as fluorescent activated cell sorting (FACS), T1 gadolinium-enhanced magnetic resonance imaging (MRI), FITC-albumin leakage assays, microvessel isolation, western blotting and immunofluorescent microscopy, we show that *in vivo* stimulation of CNS infiltrating antigen-specific CD8 T cells initiates astrocyte activation, alteration of BBB tight junction proteins and increased CNS vascular permeability in a non-apoptotic manner. Using the aforementioned techniques, we found that despite having similar expansion of CD8 T cells in the brain as wildtype and Fas Ligand deficient animals, perforin deficient mice were resistant to tight junction alterations and CNS vascular permeability. To our knowledge, this study is the first to demonstrate that CNS infiltrating antigen-specific CD8 T cells have the capacity to initiate BBB tight junction disruption through a non-apoptotic perforin dependent mechanism and our model is one of few that are useful for studies in this field. These novel findings are highly relevant to the development of therapies designed to control immune mediated CNS vascular permeability.

## Introduction

Disruption of the BBB is a hallmark feature of immune-mediated neurological disorders as diverse as viral hemorrhagic fevers, cerebral malaria and acute hemorrhagic leukoencephalitis [1]–[6]. While immune-mediated CNS vascular permeability is a likely contributor to pathology in neurologic disease, the role of CD8 T cells in BBB breakdown under inflammatory conditions remains largely undefined.

Studies using EM have determined that the healthy, intact neurovascular unit (NVU) consists of cerebral endothelial cells (CECs), basal lamina, astrocytic endfoot processes, pericytes and neurons [7]. Among these cell types, pericytes and astrocytes have the most direct interaction with vasculature. Astrocytic endfeet are in contact with 90% of abluminal CECs [4], [8]. *In vitro* and *in vivo* studies support a role for astrocytes in controlling BBB maintenance and regulation through their interaction with CECs [9]–[13]. CEC tight junctions are composed of transmembranous proteins including occludin, claudin-5 and the cytoplasmic proteins zona occludens 1, 2 and 3 (ZO-1, ZO-2, ZO-3) [3]. Consistent with *in vitro* models, CNS vascular permeability coincides with alteration of CEC tight junctions in rodent models of BBB breakdown [3], [14]–[16].

Although current models hypothesize that immune cells promote opening of the BBB in human disease, the role of CNS infiltrating antigen specific CD8 T cells in initiating vascular permeability has not been determined. Expansion and global activation of CD8 T cells has been observed in cases of viral hemorrhagic fever, suggesting that these cells may play an important role in vascular permeability [17]–[19]. To study the interaction between CD8 T cells and the NVU under neuro-inflammatory conditions, our laboratory has developed a novel mouse model of CNS vascular permeability using a variation of the Theiler's murine encephalomyelitis virus (TMEV) model commonly used to study multiple sclerosis [20]–[22]. Seven days post TMEV infection, there is a massive expansion of CNS infiltrating CD8 T cells specific for a 10 amino acid TMEV peptide, VP2_121–130_, presented in the context of the D^b^ class I molecule [23], [24]. Intravenous administration of 0.1 mg of VP2_121–130_ peptide seven days post infection results in severe CNS vascular permeability in C57BL/6 mice, leading to mortality within 48 hours [20]. In previous studies, we observed that major histocompatibility complex (MHC) class II^−/−^ (CD4 T cell deficient), IFN-γR^−/−^, TNF-α^−/−^, TNFR1^−/−^, TNFR2^−/−^, and TNFR1/TNFR2 double knockout mice were susceptible to this fatal syndrome. We also determined that inhibition of interleukin-1 and lymphotoxin-β activity did not protect mice from becoming moribund. In contrast, perforin deficient mice did not become moribund in this system [20]. These data indicated that obvious candidate cytokines as well as CD4 T cell responses were not necessary for the development of this fatal syndrome. Therefore, the objective of this study was to determine the extent immune effector proteins utilized by CD8 T cells, perforin and FasL, contributed to CNS vascular permeability and the resultant fatal syndrome.

In this paper, we present evidence that antigen specific CD8 T cells have the capacity to initiate changes to the NVU as measured by astrocyte activation and alteration of CEC tight junction proteins. Our findings confirm that CD8 T cells specific for the D^b^:VP2_121–130_ epitope initiate CNS vascular permeability and morbidity in C57BL/6 mice. Furthermore, we have determined that this process is perforin dependent.

## Results

### TMEV infected C57BL/6, C57BL/6 Prf1^−/−^ and C57BL/6 FasL^−/−^ mice have comparable acute CD8 T cell responses

**Figure 1:**
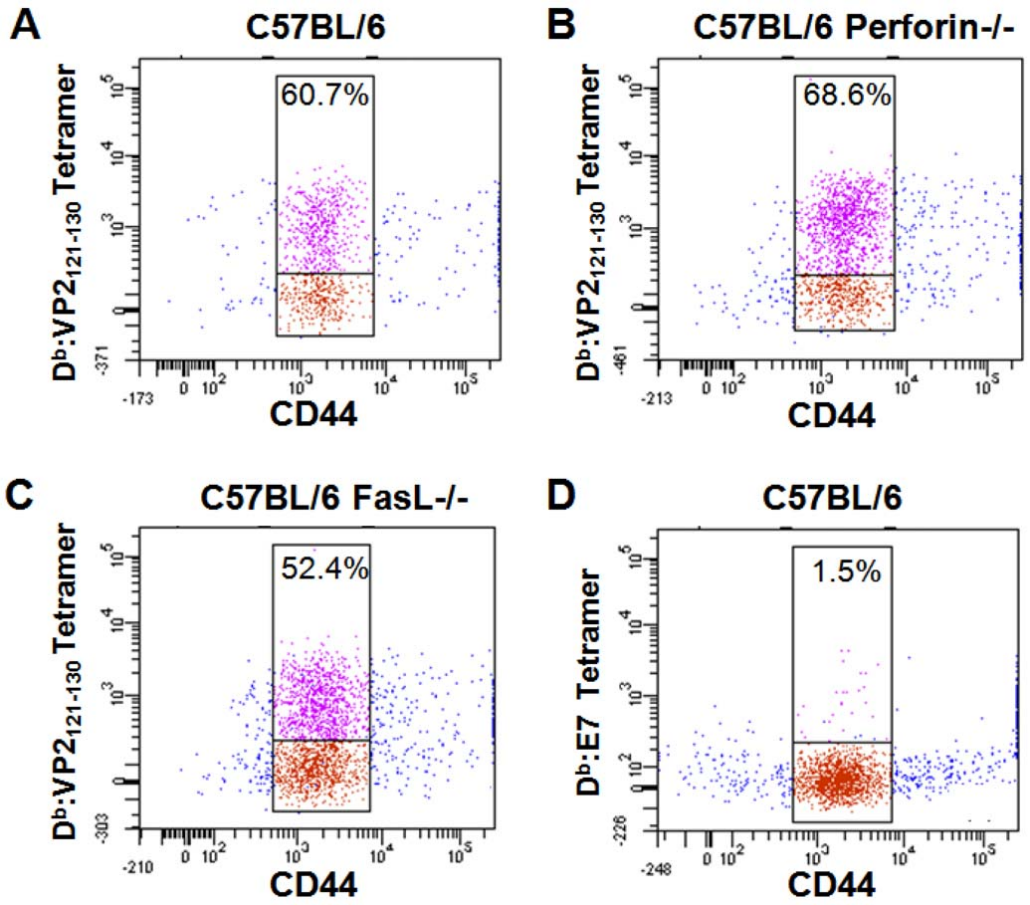
D^b^:VP2_121–130_ epitope dominance among CNS infiltrating CD8 T cells isolated from the brain seven days post-TMEV infection. Shown are brain infiltrating CD8+ cells isolated from (A) C57BL/6 mice, (B) C57BL/6 Prf1^−/−^ mice and (C) C57BL/6 FasL^−/−^ mice were gated and analyzed for CD44 activation marker expression and D^b^:VP2_121–130_ tetramer staining. In (D), C57BL/6 CD8+ cells were stained with negative control D^b^:E7 tetramer and anti-CD44. Upper gates denote percentage of CD44+ cells that stain with D^b^:VP2_121–130_ or D^b^:E7 tetramer.

To assess the CD8 T cell response of immune effector protein deficient mice during acute TMEV infection, we performed flow-cytometric analysis of lymphocytes isolated from the brain. Isolated CNS infiltrating lymphocytes from 7 day TMEV infected C57BL/6 (n = 4), C57BL/6 Prf1^−/−^ (n = 4) and C57BL/6 FasL^−/−^ (n = 4) mice were stained with antibody to CD8, CD44 and D^b^:VP2_121–130_ tetramer or irrelevant D^b^:E7 tetramer control. Using D^b^:VP2_121–130_ tetramer staining, we determined that in the brains of C57BL/6, C57BL/6 Prf1^−/−^ and C57BL/6 FasL^−/−^ mice, approximately 50–70% of CD44+ CD8+ T cells were specific for the D^b^:VP2_121–130_ epitope regardless of strain genotype (Figure 1A–C). Irrelevant D^b^:E7 tetramer did not appreciably stain CD8 T cells (Figure 1D) [23]. We did not observe overt differences in the number of isolated immune cells or frequencies of CD8+ cells among the three genotypes (data not shown). These results demonstrated that the expansion of CNS infiltrating D^b^:VP2_121–130_ epitope specific CD8 T cells was not markedly different between C57BL/6, C57BL/6 Prf1^−/−^ and C57BL/6 FasL^−/−^ mice post TMEV infection.

### Vascular permeability is dependent on perforin expression

**Figure 2:**
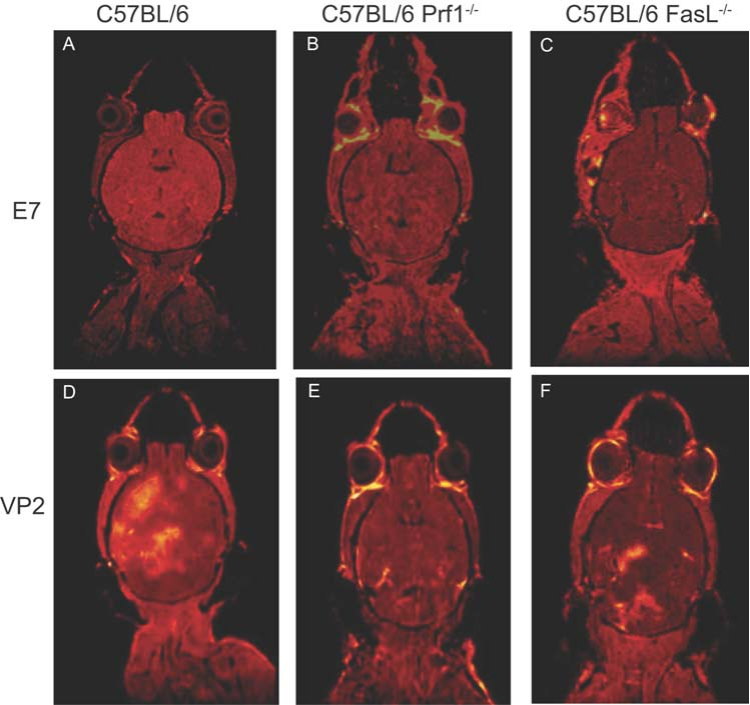
Vascular permeability following administration of VP2_121–130_ peptide is dependent on perforin expression. Analysis of blood brain barrier disruption in a representative C57BL/6, C57BL/6 Prf1^−/−^ and C57BL/6 FasL^−/−^ mouse was analyzed with T1 weighted gadolinium-enhanced MRI. Shown is the extent of gadolinium leakage in C57BL/6 (A, D), C57BL/6 Prf1^−/−^ (B, E), and C57BL/6 FasL^−/−^ (C, F) mice 24 hours post administration of control E7 peptide or VP2_121–130_ peptide. Upper panels (A–C) denote E7 peptide administration, lower panels (D–F) denote VP2_121–130_ peptide administration.

**Figure 3:**
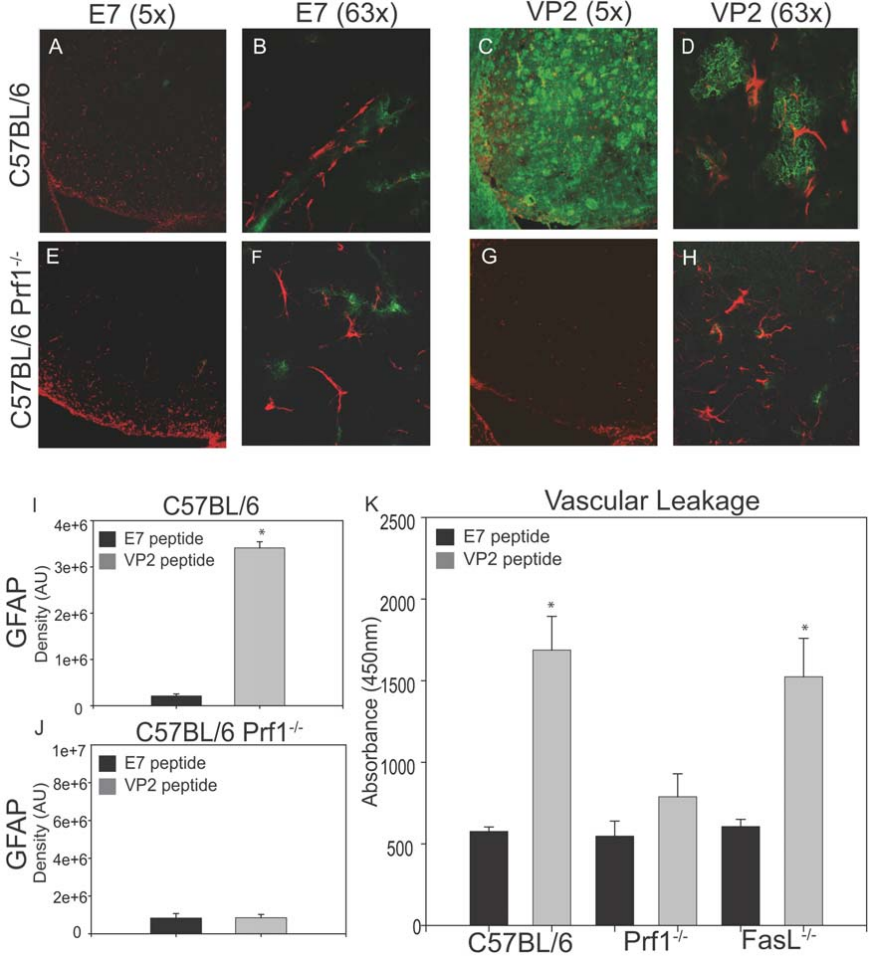
Vascular permeability and astrocyte activation following administration of VP2_121–130_ peptide is dependent on perforin expression. Tissue sections obtained from the brains of each animal were analyzed for astrocyte activation and vascular permeability as measured by leakage of FITC albumin into the CNS and expression of GFAP. Shown is GFAP expression and the extent of FITC-Albumin leakage in the striatum of E7 control or VP2_121–130_ peptide administered animals. Animals represented are (A) C57BL/6, E7 treatment, 5×, (B) C57BL/6, E7 treatment, 63×, (C) C57BL/6, VP2_121–130_ treatment, 5×, (D) C57BL/6, VP2_121–130_ treatment, 63×, (E) C57BL/6 Prf1^−/−^, E7 treatment, 5×, (F) C57BL/6 Prf1^−/−^, E7 treatment, 63×, (G) C57BL/6 Prf1^−/−^, VP2_121–130_ treatment, 5×, (H) C57BL/6 Prf1^−/−^, VP2_121–130_ treatment, 63×, Western blot analysis of GFAP expression was performed on whole brain lysates isolated from (I) C57BL/6 and (J) C57BL/6 Prf1^−/−^ mice administered control E7 peptide or VP2_121–130_ peptide. (K) Whole brain lysates obtained from C57BL/6, C57BL/6 Prf1^−/−^ and C57BL/6 FasL^−/−^ mice administered control E7 peptide or VP2_121–130_ peptide were analyzed for FITC albumin leakage using a fluorescent plate reader at 488 nm excitation and 525 nm emission.

We previously reported that C57BL/6 mice were susceptible to becoming moribund following administration of VP2_121–130_ peptide seven days post TMEV infection. This peptide induced a fatal syndrome was characterized by extensive vascular permeability [20] We also determined in this study that C57BL/6 Prf1^−/−^ mice were resistant to becoming moribund, implying an important role for effector molecules in mediating pathology following induction of CD8 T cell mediated CNS vascular permeability. To determine the extent of CNS vascular permeability in C57BL/6 Prf1^−/−^ mice, we employed *in vivo* high field strength small animal magnetic resonance imaging (MRI) to assess BBB integrity in the brains of C57BL/6, C57BL/6 Prf1^−/−^ and C57BL/6 FasL^−/−^ mice, 24 hours post administration of VP2_121–130_ or mock E7 peptide (Figure 2). Using T1-gadolinium enhanced MRI we determined that C57BL/6 (Figure 2A, D) and C57BL/6 FasL^−/−^ (Figure 2C, F) mice had extensive vascular permeability in the brain. In contrast, C57BL/6 Prf1^−/−^ mice had preserved vascular integrity as indicated by a lack of gadolinium enhancement in the brain following VP2_121–130_ peptide administration (Figure 3B, E).

MRI provided a qualitative analysis of vascular permeability in C57BL/6, C57BL/6 Prf1^−/−^, and C57BL/6 FasL^−/−^ mice. To enable a statistical analysis to test the hypothesis that C57BL/6 Prf1^−/−^ mice were resistant to CNS vascular permeability using a larger group of animals, we developed a liquid assay to analyze whole brain lysates. Intravenous injection of FITC-albumin is a method of quantifying CNS vascular permeability breakdown, as albumin does not readily cross the BBB under normal conditions[25]. This technique enabled us to quantify CNS vascular permeability in brain homogenates with an immunofluorescent plate reader. C57BL/6, C57BL/6 Prf1^−/−^, and C57BL/6 FasL^−/−^ mice were administered E7 control peptide or VP2_121–130_ peptide to induce CD8 T cell mediated vascular permeability. FITC-albumin was then administered intravenously to E7 and VP2_121–130_ peptide treated C57BL/6, C57BL/6 Prf1^−/−^, and C57BL/6 FasL^−/−^ mice and allowed to circulate for one hour prior to brain harvest. Harvested brains were homogenized and permeabilized to determine the extent of FITC-albumin leakage into the brain. We observed significant increases in FITC-albumin leakage in C57BL/6 (E7 n = 4 mice, VP2 n = 3 mice) (P<0.01) and C57BL/6 FasL^−/−^ (E7 n = 4 mice, VP2 n = 3 mice) (P<0.01) but not C57BL/6 Prf1^−/−^ (E7 n = 4 mice, VP2 n = 4 mice) mice 24 hours post VP2_121–130_ peptide administration (Figure 3K). These data demonstrated that perforin but not FasL expression was necessary for VP2_121–130_ peptide induced CNS vascular permeability.

### Astrocyte activation is dependant on perforin expression

Astrocytes have been demonstrated to regulate BBB integrity *in vitro* and *in vivo* [9], [10], [13]. Therefore, we assessed astrocyte activation in C57BL/6 and C57BL/6 Prf1^−/−^ mice 24 hours post administration of VP2_121–130_ or mock E7 peptide. As demonstrated by microscopy, brains of C57BL/6 mice showed glial fibrillary acidic protein (GFAP) expression by astrocytes in regions with FITC-albumin leakage (Figure 3A–D). In contrast, C57BL/6 Prf1^−/−^ mice lacked an increase in GFAP expression with VP2_121–130_ peptide treatment (Figure 3E–H). We assessed GFAP protein levels in C57BL/6 and C57BL/6 Prf1^−/−^ using western blot analysis of whole brain lysates. These data showed a significant increase in GFAP expression in C57BL/6 treated with VP2_121–130_ peptide (n = 3 mice) than with E7 control (n = 4 mice) (P<0.01) (Figure 3I). C57BL/6 Prf1^−/−^ treated with VP2_121–130_ peptide (n = 4 mice) did not have a significant increase in GFAP expression when compared to E7 control (n = 4 mice) (Figure 3J). We determined from these experiments that perforin is necessary for upregulation of GFAP following VP2_121–130_ peptide administration.

### Rapid NVU alterations precede peak increases in vascular permeability

**Figure 4:**
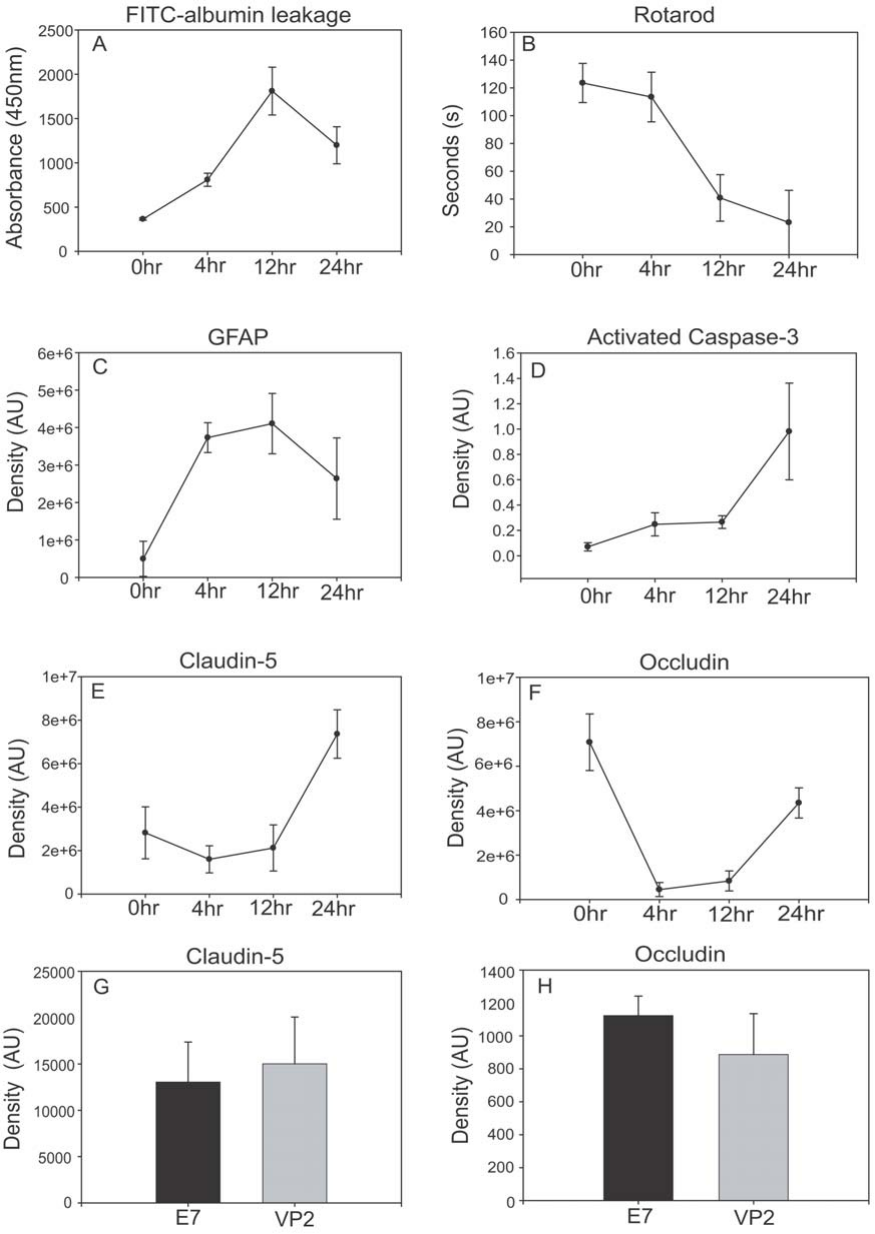
Astrocyte activation and occludin degradation occur prior to vascular permeability and motor deficits. At 0, 4, 12 and 24 hours post administration of VP2_121–130_ peptide, C57BL/6 mice were analyzed for (A) FITC-Albumin leakage in whole brain, (B) motor performance using rotarod analysis, (C) GFAP expression in whole brain, (F) Occludin protein levels in isolated brain microvessels, (E) Claudin-5 protein levels in isolated brain microvessels and (D) active caspase-3 protein levels in isolated brain microvessels. Mice were analyzed with rotarod analysis (B) just prior to harvesting CNS tissue. Also shown are 24 hours post-administration of control E7 peptide or VP2_121–130_ peptide in C57BL/6 Prf1^−/−^ mouse tight junction protein levels of (G) claudin-5, (H) occludin.

In the above experiments, we determined that administration of the immunodominant VP2_121–130_ peptide post TMEV infection resulted in astrocyte activation and vascular permeability. We next performed a time course experiment to monitor the order in which astrocyte activation, alteration of cerebral endothelial tight junction protein levels, CNS vascular permeability and functional deficit occurred. Using western blot analysis, we examined GFAP protein (Figure 4C), occludin and claudin-5 protein (Figure 4E, F) and active capase-3 protein (Figure 4D) at 0, 4, 12, and 24 hours post VP2_121–130_ peptide administration in C57BL/6 mice. We also examined FITC-albumin leakage (n = 4 at 0,4,12,24 hours) (Figure 4A) and rotarod performance (Figure 4B) at 0 hours (n = 16), 4 hours (n = 12), 12 hours (n = 8) and 24 hours (n = 4) following administration of VP2_121–130_ peptide. Astrocyte activation and tight junction protein alterations were observed as early as 4 hours post administration of VP2_121–130_ peptide (Figure 4C, E, F). CNS vascular permeability was greatest at 12 hours post- VP2_121–130_ peptide administration as assessed by FITC-albumin leakage. Ability to negotiate the rotarod decreased at 12 hours and continued to decline until 24 hours. To determine if CECs were undergoing apoptosis during peak levels of vascular permeability, active caspase-3 protein levels were determined in the microvessel lysates. We did not observe an overt increase in active caspase-3 until 24 hours in these lysates (n = 4 at 0, 4, 12, 24 hours), suggesting that activation of apoptosis does not occur until after peak levels of permeability. Unlike perforin competent C57BL/6 mice, C57BL/6 Prf1^−/−^ mice maintained tight junction expression levels of occludin and claudin-5 similar to controls for 24 hours post administration of VP2_121–130_ peptide (Figure 4G, H). These data demonstrate that perforin expression is necessary for tight junction alterations following administration of VP2_121–130_ peptide. These results also indicate that astrocyte activation and tight junction alterations develop prior to peak levels of vascular permeability and functional impairment in this model.

## Discussion

The observation that CD8 T cells contribute to vascular permeability and utilize class I molecules to traffic to the CNS extends beyond the TMEV model put forth in this study [26]. Clinical studies of human cerebral malaria have shown that tight junction proteins are altered during infection similar to what we observe in our model [6]. Animal models of cerebral malaria have also presented strong evidence that CD8 T cells are involved in neuropathology [27]. Among the human viral hemorrhagic fevers, dengue hemorrhagic fever (DHF) has been the most widely studied. In DHF, considerable CD8 T cell activation, expansion and apoptosis can be measured in the blood of DHF patients during the peak of disease [17]. Additional epidemiological studies among ethnic Thais have determined there is a genetic predisposition to DHF which is linked to specific class I human leukocyte antigen alleles [19]. Human leukocyte antigen class I alleles contributing to a genetic predisposition have also been observed in Haantavirus pulmonary syndrome among residents of the Southwestern United States [18], [28].

Previously, we reported that the majority of CNS infiltrating CD8 T cells recognize the VP2_121–130_ peptide presented in the context of the D^b^ class I molecule in seven day TMEV infected C57BL/6 mice [23]. In ensuing studies designed to manipulate the D^b^:VP2_121–130_ epitope specific CD8 T cell response, we determined that these antigen specific CD8 T cells could initiate a fatal condition characterized by vascular permeability upon administration of the VP2_121–130_ peptide during acute TMEV infection [20]. Specific removal of this antigen specific population of CD8 T cells protected mice from becoming moribund, demonstrating the critical necessity of these cells in initiating this fatal condition [20], [24]. Using specific knockout mice and soluble receptor techniques, we determined that this fatal condition was not mediated by obvious cytokine candidates, including TNFα, lymphotoxin-β and interleukin-1 [20].

We initially reported that perforin deficient mice had autofluorescence in the hippocampal region of the brain and that this was presumably due to vascular leakage of blood into the brain [20]. This was an unexpected finding given that perforin deficient mice were protected from becoming moribund following VP2_121–130_ peptide administration. The current study was designed to revisit this unexpected result using superior technology, including MRI, established vascular permeability assays, and high-powered microscopy. Using these established techniques to analyze BBB disruption, we have determined that perforin is necessary for CNS vascular permeability in this model and that autofluorescence is not an appropriate method of analysis. The results obtained in the current study are also consistent with preservation of functional ability in perforin deficient mice after administration of VP2_121–130_ peptide [20]. Furthermore, the use of autofluorescence to monitor CNS vascular permeability is not established in the literature. One possibility is that the methodology used in the prior study played an important role in the autofluorescence seen in the previous study [20]. Mouse brains were fixed in 4% paraformaldehyde and cut with a vibratome. In the present study, we used *in vivo* MRI (figure 2), fresh frozen tissue homogenates for FITC-albumin leakage assays (figure 3) and ethanol fixation of fresh frozen tissue sections to visualize FITC-albumin leakage (figure 3). These assays demonstrate that C57BL/6 Prf1^−/−^ mice are resistant to CNS vascular permeability in this model, suggesting a different biological process was responsible for autofluorescence in the previous study. It has been reported that oxidative damage to erythrocytes can result in increased autofluorescence [29], [30]. It is therefore possible that administration of VP2_121–130_ peptide initiates a perforin-independent mechanism that results in oxidative activity in the CNS that may have been misconstrued as vascular permeability. Nevertheless, it is important to note that autofluorescence is not a reliable method for measuring vascular leak under neuroinflammatory conditions.

In light of the discrepancy between past and present findings, we further sought to define the importance of perforin expression in promoting vascular permeability and functional impairment by crossing mice heterozygous for perforin (F1) with C57BL/6 Prf1^−/−^ mice. The resulting offspring were 50% heterozygous for the perforin allele and 50% perforin deficient. Consistent with the results of experiments put forward in this paper, perforin heterozygosity predisposed these animals to undergo vascular permeability shown by FITC-albumin leakage (P<0.01) and functional impairment on the rotarod (P<0.01) when compared to perforin deficient littermate controls (data not shown). This experiment demonstrates that only one perforin allele is necessary to induce this syndrome and minimizes the likelihood of other contributing alleles on the C57BL/6 Prf1^−/−^ mouse background that may have resulted through genetic drift or incomplete crossing from the 129 svlm mouse background.

To understand the state of tight junction proteins in our model of CNS vascular permeability we utilized a well established method of rodent brain microvessel isolation [31]–[33]. Microvessel isolation from brain tissue enabled the analysis of BBB tight junction protein levels in the CNS. Assessment of tight junction protein levels in brain microvessels isolated from C57BL/6 and C57BL/6 Prf1^−/−^ mice demonstrated that perforin was necessary for alteration of BBB tight junction protein levels in C57BL/6 mice. In these assays, we observed a decrease in occludin protein as early as 4 hours post VP2_121–130_ peptide administration and an increase in claudin-5 by 24 hours in C57BL/6 mice (Figure 4). These data are significant because decreased transendothelial electric resistance indicative of increased paracellular permeability has been observed in *in vitro* BBB models with compromised occludin expression [34]. Furthermore, claudin-5 has been hypothesized to play a role in tight junction formation and is predominantly expressed in CECs rather than nonvascular CNS tissue [35], [36]. Using the same microvessel isolation technique in a murine model of chronic inflammatory pain also resulted in decreased occludin and increased claudin-5 expression when BBB breakdown was initiated [37]–[39]. Although our model of CNS vascular permeability is initiated by CNS infiltrating antigen specific CD8 T cells through a process dependent on perforin, our data supports these previous findings that alteration of tight junction protein levels is concurrent with BBB disruption.

We have determined through preliminary studies, that low levels of vascular permeability are occurring as a result of TMEV infection in the CNS (data not shown). This preliminary work suggests that intravenously injected VP2 peptide has access to the CNS through low levels of BBB permeability potentially resulting in heightened activation of CNS infiltrating CD8 T cells in this model. We previously reported a significant reduction in D^b^:VP2_121–130_ epitope specific CD8 T cells, one day following VP2 peptide administration in both C57BL/6 and 129 SvIm strains of mice, demonstrating a rapid effect of VP2 peptide on CNS infiltrating CD8 T cell responses [20]. Additionally, the T1 gadolinium-enhanced MRI analysis put forth in this study further supports the capacity of CNS infiltrating CD8 T cells in altering the BBB. We consistently observe that the hemisphere intracranially injected with TMEV has more pronounced vascular permeability compared to the contralateral side (Figure 2 and data not shown). This specific regional enhancement near areas of virus injection suggests that high levels of viral replication, antigen processing and antigen presentation to CD8 T cells in the CNS tissue predisposes specific regions of the brain to be susceptible to vascular permeability. This MRI study coupled with our previous work which determined that the majority of D^b^:VP2_121–130_ epitope specific CD8 T cells are predominantly found in the brain 7 days post TMEV infection and not in peripheral lymphoid compartments, supports the concept that active engagement of T cell receptor with antigen in the CNS is a likely contributor to vascular permeability following administration of intravenous VP2_121–130_ peptide [20], [23]. Previous demonstration of normal hematocrit levels and a lack of pathology in peripheral organs in this model further supports such a hypothesis [20].

C57BL/6 Prf1^−/−^ mice have negligible astrocyte activation, normal levels of tight junction proteins and the absence of CNS vascular permeability following administration of VP2_121–130_ peptide administration. These data demonstrate a critical role for perforin in disruption of the NVU which results in CNS vascular permeability. We hypothesize that CNS infiltrating CD8 T cells are hyper-stimulated by the presence of excess VP2_121–130_ peptide antigen presented on D^b^ class I molecules in the CNS. These D^b^:VP2_121–130_ epitope specific CD8 T cells then utilize perforin to deliver inflammatory components which ultimately result in activation of cellular components of the NVU, leading to rapid alteration of BBB tight junction proteins. This results in CNS vascular permeability and decreased ability to negotiate the rotarod (Figures 2, 3).

An alternative model is that CD8 T cells destroy vascular CECs directly through direct perforin-mediated killing. Studies in murine cerebral malaria have found that perforin deficient mice are resistant to CEC damage, as assessed by active caspase-3 staining. The authors demonstrate that caspase-3 activation in CECs occurred simultaneously with edema and petechial hemorrhage and that this process was perforin dependent [27]. We also observed caspase-3 activation in microvessel isolates at 24 hours post VP2_121–130_ peptide administration when the C57BL/6 mice were moribund. However, the onset of FITC-albumin leakage occurs much earlier, peaking at 12 hours post administration of VP2_121–130_ peptide which is considerably earlier than observable active caspase-3 in microvessel isolates (Figure 4). Our kinetic analysis therefore does not readily support a mechanism in which caspase-3 mediated apoptosis is the initiator of CNS vascular permeability. Furthermore, while we observed decreases in microvessel occludin following VP2_121–130_ peptide administration, Claudin-5 levels dramatically increased. These data suggest that CECs remained viable and capable of protein expression while peak permeability was occurring (Figures 4). Our analysis of tight junction proteins and active caspase-3 protein in microvessel isolates supports a model in which perforin mediates vascular permeability through a mechanism that is not directly apoptotic to the vasculature or utilizes a pathway that does not involve caspase-3 activation. A non-apoptotic role for perforin has been previously implied in studies demonstrating the capacity of CD8 T cells to control Herpes simplex virus replication in ganglionic neurons through a mechanism that does not result in apoptosis [40], [41]. Extending this observation to other CNS cell types enables one to hypothesize that perforin could potentially deliver inflammatory factors to CNS cell types, including cellular components of the neurovascular unit, without initiating apoptosis.

## Methods and Materials

### Animals

C57BL/6J, C57BL/6-Prf1 (Prf1^−/−^, Perforin deficient), B6Smn.C3-Tnfsf6 (FasL^−/−^) mice were obtained from Jackson laboratories at 6 weeks of age. All experiments were approved by the IACUC of University of Cincinnati.

### Induction of CNS vascular permeability

CNS vascular permeability was induced as described previously [20]. Briefly, all mice were infected intracranially with 2×10^6^ PFU Daniel's strain of TMEV. 7 days post-TMEV infection, mice were injected intravenously with 0.1 mg VP2_121–130_ (FHAGSLLVFM) or mock control E7 (RAHYNIVTF) peptide (Genscript) [20].

### Rotarod

Animals were placed on the Rotamex-5 rotarod apparatus (Columbus Instruments) increasing from 5–40 RPMs over 7 minutes. This behavioral assay has been well established as a means to assess overall functional ability in mice ([42], [43]. For multiple time points rotarod score is represented as seconds on rotarod at 0,4,12 and 24 hours post-VP2_121–130_ peptide administration (Figure 4).

### FITC-albumin Permeability Assay

Mice were injected intravenously with 10 mg FITC-albumin (Sigma #A9771) 23 hours after VP2_121–130_ peptide administration and brains were harvested at 24 hours. Brains were cut using a cryostat at −20°C. All sections not mounted on slides were used to make homogenates as described in Western blots method (minus brainstem). Homogenates were read on a fluorescent plate reader at 488 nm excitation and 525 nm emission. Data was collected using SpectraMax software (Molecular Devices).

### Western Blots

Brain tissue samples were lysed in radioimmunoprecipitation assay (RIPA) buffer [10 mmol/L Tris, 140 mmol/L NaCl, 1% Triton X-100, 1% Na deoxycholate, 0.1% SDS and protease inhibitor cocktail (Pierce #78410) pH 7.5] and centrifugated for 15 min at 10,000 RPM. Samples were normalized using the BCA protein assay (Pierce #23223) and prepared using the method of Laemmli [44]. For GFAP detection, 10 ug of protein was loaded per well on 4–20% precise protein gels (Pierce #25244) with BupH Tris-HEPES-SDS running buffer (Pierce #28398). Gels were transferred onto Immun-Blot PVDF membranes (Biorad #162-0177) using Tris transfer buffer [400 mmol/mL Tris base, 70 mmol/mL glycine, 10% methanol]. Mouse anti-GFAP (1∶1000 BD Pharmingen #556329) and goat anti-mouse IgG conjugated to horseradish peroxidase were used to detect GFAP (Sigma #A3682). Western blot films were analyzed by densitometry using Scion Image software (Scion Corp). Background was subtracted from each band and data is expressed in arbitrary units (AU).

### Microvessel isolation and tight junction, active caspase-3 western blots

Microvessel isolation based on protocol previously described by Brooks et al. [33]. Briefly, hemispheres ipsilateral or contralateral to the intracranial TMEV injection site were homogenized in microvessel isolation buffer, 26% dextran was added and samples were centrifuged for 10 minutes at 6874 RPM. Supernatants were discarded and pellet was resuspended in microvessel isolation buffer. Samples were centrifuged at 5010 RPM (Sorvall Superspeed SS-34 rotor) for 10 minutes. Pellets were resuspended in 6 M urea and digested overnight at 4°C. Protein concentration was determined using a BSA standard curve and 4 ug or 8 ug was added per well as described previously. For tight junction analysis, blots were probed using rabbit anti-occludin, claudin-5 (Zymed) at 1∶500. Goat anti-rabbit conjugated to horseradish peroxidase (Santa Cruz #sc 2004) was used to detect primary antibody. For active Caspase-3 detection, 30 ug protein was loaded per well on 4–20% Tris-HCL Criterion precast gels (Bio-Rad #345-0033). Gels ran in Tris-SDS running buffer and were transferred onto Immun-Blot PVDF membranes (Biorad #162-0177) using Tris transfer. Rabbit anti-active Caspase-3 (BD Biosciences #557038) and goat anti-rabbit conjugated to horseradish peroxidase (Santa Cruz #sc 2004) was used to detect active caspase-3 protein. Western blot films were analyzed by densitometry as described above.

### Immunofluorescent microscopy

Tissue sections from FITC-albumin injected mice were fixed in −20°C 95% ethanol at 4°C for 15 minutes and washed with repeated changes in .01 M PBS for 2 hours. Sections were blocked in 10% normal goat serum (in 0.1 M PBS+.03% Triton X-100) for 1 hour. Sections were incubated in mouse anti-GFAP conjugated to cy-3 (Sigma C-9205) overnight at 4°C in humid chamber. Sections were washed in 0.1 M PBS for 1 hour and air dried for 1.5 hours. Fluoromount (Southern Biotech 0100-01) was used to mount coverslips. Sections were imaged at room temperature at 5× and 63× using the Orca ER Zeiss upright microscope. Images were captured using Orca-ER CCD camera and Metamorph Software (Molecular Devices Corporation) at the Center for Biological Microscopy at University of Cincinnati, College of Medicine.

### Flow cytometry

Seven days post TMEV infection, brain-infiltrating lymphocytes were isolated from mouse brain through collagenase digestion and a percoll gradient as previously described [45]. Allophycyanin (APC) D^b^:VP2_121–130_ tetramer and APC D^b^:E7 tetramer were constructed as previously published and used in conjunction with anti-CD8-FITC (BD #553031) and anti-CD44-PE (BD #553134) [23]. Samples were read on a BD LSR II flow cytometer (Becton Dickinson) and analyzed with BD FACS Diva 6.0 software (BD Biosciences). In all panels, the CD8+ T cell population was gated and analyzed for CD44+ (activation) and D^b^: VP2_121–130_ or D^b^:E7 tetramer.

### Magnetic Resonance Imaging

MRI acquisition was performed in a horizontal bore Bruker 7 Tesla microimaging system. Volume acquisition of the entire mouse brain was performed pre- and post-gadolinium contrast dye injection using a T1 weighted spin echo sequence (TR200ms, TE10ms, FOV: 4×2.5×2.5 cm, matrix: 256×128×128, NEX 2) to determine the overall volume of gadolinium enhancing areas (areas with increased BBB permeability).

### Statistical Analysis

Mean and standard error values for rotarod scores, FITC-albumin quantification and western blot quantification were calculated using software program SigmaStat(SYSTAT software Inc). Bar graphs with standard error values were plotted on software program SigmaPlot (SYSTAT software Inc). To determine significance between groups, a student's t-test was performed between E7 and VP2_121–130_ within the same strain using SigmaPlot.
